# The effect of exercise interventions on mental health in children and adolescents with attention-deficit/hyperactivity disorder: a meta-analysis

**DOI:** 10.3389/fpsyg.2026.1748777

**Published:** 2026-02-16

**Authors:** Zhou Shenning, Hou Yaoqi, Shi Wenying, Song Xiangqin

**Affiliations:** School of Physical Education and Sports, Beijing Normal University, Beijing, China

**Keywords:** attention deficit hyperactivity disorder, exercise intervention, exercise therapy, mental health, meta-analysis

## Abstract

**Background:**

Mental health issues such as anxiety and depression are common comorbidities in individuals with Attention-Deficit/Hyperactivity Disorder, exacerbating functional impairments. Exercise interventions show promise as a non-pharmacological approach, but their specific efficacy on mental health in the ADHD population remains controversial. This study aimed to evaluate the effect of exercise interventions on mental health in children and adolescents with ADHD and to explore potential moderating factors.

**Methods:**

This meta-analysis adhered to the PRISMA guidelines and was registered with PROSPERO (CRD420251162082). We systematically searched five electronic databases, including PubMed and Web of Science, from inception until October 2025. Randomized or quasi-randomized controlled trials evaluating the impact of exercise interventions on mental health (depression, anxiety, emotion regulation) in individuals with ADHD were included. Study quality was assessed using the Cochrane Risk-of-Bias tool (ROB 2) and the PEDro scale. The standardized mean difference (SMD) was calculated as the effect size using random-effects models. Data analysis was performed using Stata 18.0.

**Results:**

Eighteen studies were included in the meta-analysis. The results demonstrated that exercise interventions had a significant positive effect on improving depressive symptoms (SMD = 0.42, 95% CI: 0.13–0.71) and emotion regulation ability (SMD = 0.47, 95% CI: 0.06–0.92) in individuals with ADHD. Exercise interventions also significantly alleviated anxiety symptoms (SMD = 0.43, 95% CI: 0.006–0.84). Exploratory subgroup analyses suggested that mind-body exercises (e.g., yoga) may be associated with particularly larger improvements in depression (SMD = 0.46) and anxiety (SMD = 0.80), while moderate-intensity exercise appeared to show favorable efficacy for depression (*SMD* = 0.54). Meta-regression indicated a potential, though statistically non-significant, trend for the effect on depression to diminish with increasing age.

**Conclusion:**

This meta-analysis provides evidence supporting exercise interventions, particularly mind-body and moderate-intensity exercise, as an effective adjunctive approach for improving mental health in individuals with ADHD. Exploratory findings suggest that mind-body and moderate-intensity exercises may offer specific benefits; however, further high-quality studies are warranted to confirm these specific parameters. The findings provide preliminary insights for developing individualized exercise prescriptions, emphasizing the need for caution in generalizing specific exercise types and intensities.

**Systematic review registration:**

https://www.crd.york.ac.uk/prospero/, identifier CRD420251162082.

## 1 Introduction

Attention-Deficit/Hyperactivity Disorder (ADHD) is a highly prevalent neurodevelopmental disorder, particularly common among children and adolescents. With a global prevalence of approximately 7%, ADHD poses a substantial and increasing disease burden worldwide (de la Pena et al., 2020). The core clinical manifestations of ADHD-age-inappropriate inattention, hyperactivity, and impulsivity (Mechler et al., 2022)—are frequently accompanied by significant emotional dysregulation, including symptoms of anxie ty and depression (Caldwell and Welton, 2016). A considerable body of research indicates that such comorbidities are common in individuals with ADHD, which not only exacerbates the core symptoms and impairs social functioning but also elevates the long-term risk for other psychiatric disorders and maladaptive behaviors (Kouros et al., 2024; Martz et al., 2023; Murray et al., 2021). Consequently, identifying effective interventions to enhance the mental health of individuals with ADHD is of paramount practical importance.

Current clinical management of ADHD primarily relies on pharmacological treatments (e.g., central nervous system stimulants) and behavioral therapies (e.g., cognitive-behavioral therapy) (Veronesi et al., 2024). However, medication can be associated with adverse effects such as sleep disturbances and decreased appetite, while behavioral interventions often involve lengthy durations and face limitations in resource accessibility (Zhang et al., 2025; Chang et al., 2015). In this context, exercise intervention has emerged as a promising adjunctive approach due to its high safety profile, low cost, and ease of implementation (Sibley et al., 2022). Growing evidence suggests that exercise can positively impact both the core symptoms and the psychological state of individuals with ADHD, with effects sometimes comparable to certain medications (Dahiya et al., 2025; Fietsam et al., 2022). The potential mental health benefits of exercise are underpinned by multi-faceted mechanisms. Physiologically, exercise promotes the release of brain-derived neurotrophic factor (BDNF) and enhances neuroplasticity in key brain regions governing emotion and stress response, such as the prefrontal cortex and limbic system (e.g., hippocampus, amygdala) (Kandola et al., 2019). It also modulates the autonomic nervous system, increasing heart rate variability and thereby reducing physiological arousal to promote emotional stability (Liu et al., 2025). From a psycho-behavioral perspective, exercise provides a structured and predictable framework. The sense of achievement and mastery gained during physical activity can boost self-efficacy and counteract learned helplessness associated with chronic frustration (Biddle and Asare, 2011). Furthermore, team-based sports offer valuable opportunities for positive social interaction, fostering mental health through enhanced social support (Eime et al., 2013).

Despite its potential, findings regarding the efficacy of exercise interventions on emotional problems like anxiety and depression in ADHD remain inconsistent and controversial. While some randomized controlled trials and meta-analyses report small to moderate reductions in anxiety symptoms following moderate-to-vigorous aerobic exercise (Faraone et al., 2006; Faraone and Glatt, 2010), other studies have found non-significant effects on depressive mood, and some high -intensity training might even induce emotional fluctuations (Kriel et al., 2018). These discrepancies may be attributed to variations in exercise prescription parameters (e.g., type, intensity, frequency, duration), participant characteristics, and assessment tools used across studies.

As clinical management of ADHD increasingly emphasizes personalized and mind-body integrated interventions, clarifying the mechanisms and establishing optimized exercise protocols have become critical research priorities. However, the existing body of evidence is fragmented, and a systematic evaluation of the moderating effects of key exercise prescription parameters (e.g., type, intensity) is lacking. This gap hinders the development of precise, evidence-based guidelines for clinical practice. Therefore, this meta-analysis aims to quantify the effects of exercise interventions on different domains of mental health (depression, anxiety, emotion regulation) in children and adolescents with ADHD. Utilizing subgroup analysis and meta-regression, we investigated whether critical variablesdepresas exercise type, intensity, duration, and participant ageriablesdepression, anxiety, emotion regulation) in children and adolescents wto provide a scientific basis for developing more targeted and feasible exercise prescriptions.

## 2 Materials and methods

### 2.1 Protocol and registration

The protocol for this meta-analysis was prospectively registered on PROSPERO (International Prospective Register of Systematic Reviews; registration number: CRD420251162082). The design, conduct, and reporting of this review adhered to the PRISMA (Preferred Reporting Items for Systematic Reviews and Meta-Analyses) 2020 statement (Page et al., 2021) to ensure a rigorous and transparent methodology in literature search, study selection, data extraction, risk of bias assessment, and data synthesis, thereby enhancing the reproducibility and reliability of the findings.

### 2.2 Search strategy

A comprehensive literature search was conducted across five electronic databases: PubMed, Embase, Web of Science, Cochrane Library, and CNKI (China National Knowledge Infrastructure), covering records from their inception to October 25, 2025. Studies published in English and Chinese were included to ensure broad coverage of relevant research. The search strategy was constructed by combining Medical Subject Headings (MeSH) terms and free-text words related to three key concepts: (1) attention deficit hyperactivity disorder (ADHD), (2) exercise or physical activity interventions, and (3) mental health (MeSH, 2025). Boolean logical operators (AND, OR) were used to appropriately combine these components (MeSH, 2025).

### 2.3 Inclusion criteria

The inclusion criteria were established based on the PICOS (Participants, Interventions, Comparisons, Outcomes, Study Design) framework (Amir-Behghadami and Janati, 2020). Participants (P): Children and adolescents (under 18 years of age) with a formal diagnosis of ADHD, irrespective of gender, region, or language. Diagnosis could be based on DSM, ICD, or other recognized diagnostic criteria. Interventions (I): Any intervention primarily based on physical activity or exercise, including sports, fitness training, mind-body exercises (e.g., yoga, tai chi), and virtual reality-based exercises. There were no restrictions on the specific type, intensity, frequency, or duration. Comparisons (C): Control groups receiving no intervention, usual care, or other non-exercise interventions. Multi-arm studies were eligible if the exercise interventions differed significantly between groups in type, intensity, or modality. Outcomes (O): At least one objectively measured, standardized, and quantifiable mental health outcome, such as scores from validated scales assessing emotion regulation, depression, or anxiety. Study Design (S): Randomized controlled trials (RCTs) or quasi-RCTs that reported sufficient statistical data (e.g., means, standard deviations, sample sizes) for effect size calculation.

### 2.4 Exclusion criteria

Studies were excluded if they: (1) involved mixed populations without separately reported data for the ADHD subgroup; (2) included participants outside the child/adolescent age range without subgroup data; (3) had an intervention where the exercise component was not clearly identifiable or poorly described; (4) compared intervention and control groups where the exercise nature was not substantially different (e.g., differing only in delivery setting or personnel); (5) lacked objectively measured, standardized, and quantifiable mental health outcomes; (6) lacked sufficient statistical data even after attempting to contact the authors; or (7) were reviews, commentaries, case reports, conference abstracts, or non-peer-reviewed publications.

### 2.5 Study selection process

**FIGURE 1:**
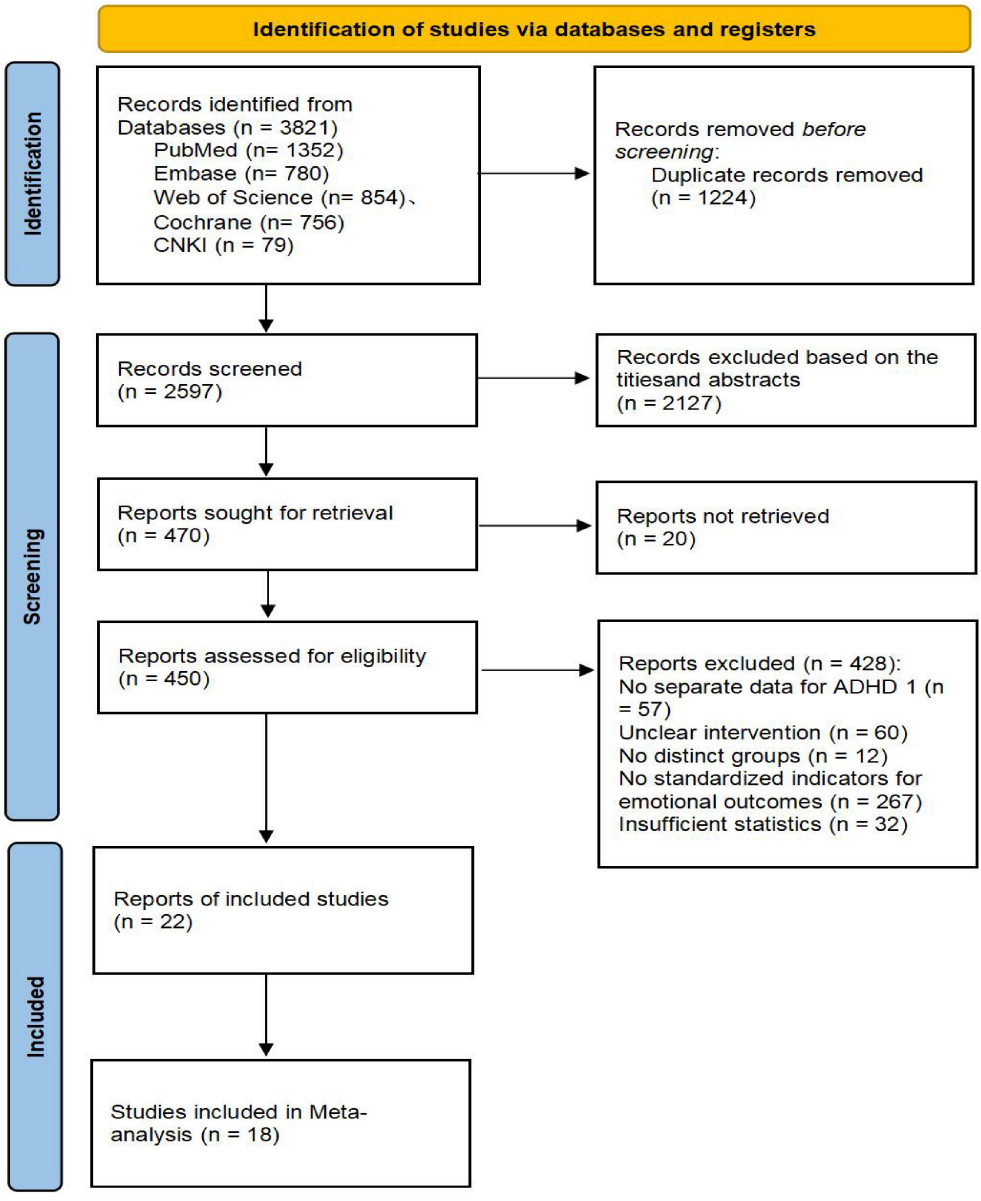
Literature screening and selection flowchart.

Two reviewers (ZSN, SWY) independently screened the titles and abstracts of references retrieved from the databases. They then assessed the full texts of potentially eligible studies, recording their decisions (0 = exclude/1 = include for full-text review) in a pre-formatted Excel spreadsheet. After removing duplicates, the reviewers independently screened the titles and abstracts of the remaining records, excluding those clearly ineligible. The full texts of the remaining articles were obtained and thoroughly evaluated. If a study was excluded, the reason was documented. If reported information was insufficient, the corresponding author was contacted via email, allowing a 2-week response period. Studies remained excluded if necessary data could not be obtained after these efforts. Any disagreements between the reviewers were resolved through discussion or by consulting a third reviewer (HYQ). The detailed selection process is illustrated in the PRISMA flow diagram (Figure 1).

### 2.6 Quality assessment of included studies

Two assessors (ZSN, SWY) independently evaluated the methodological quality of the included studies using the PEDro scale and assessed the risk of bias using the Cochrane Risk of Bias tool for randomized trials (ROB 2) (Sterne et al., 2019). The PEDro scale is an 11-item checklist designed to evaluate the methodological rigor and statistical reporting of clinical trials (the ‘eligibility criteria’ item is not scored). Each satisfied item scores 1 point. Total scores were used to categorize studies as poor (<4), fair (4–5), good (6–8), or excellent (9–10) quality. The ROB 2 tool assesses bias across five domains: randomization process, deviations from intended interventions, missing outcome data, measurement of the outcome, and selection of the reported result. Studies were classified as having “low risk,” “some concerns,” or “high risk” of bias.

### 2.7 Data extraction

To maximize accuracy and consistency, data extraction was performed independently by two researchers. Subsequently, all extracted data underwent cross-checking and verification by another team member as a further quality control measure. The extracted information included: (1) study design and basic characteristics; (2) participant demographics and clinical features (e.g., age, gender distribution); (3) detailed intervention parameters (type, frequency, intensity, duration); (4) details of the control group; (5) outcome measurement methods (Follmann et al., 1992), assessment tools, time points, and statistics necessary for effect size calculation (means, standard deviations, sample sizes). All data were entered into a structured Excel database for subsequent analysis (Drevon et al., 2017). If key data were missing, systematic attempts were made to contact the corresponding authors via email, with a reminder sent after 48 h. Studies were excluded from the final analysis if the necessary data remained unavailable.

### 2.8 Statistical analysis

Statistical analyses were performed primarily using Stata 18.0 software, encompassing effect size synthesis, subgroup analysis, sensitivity analysis, meta-regression, and publication bias assessment. To combine results from studies using different measurement tools, the effect size was expressed as the Standardized Mean Difference (SMD) with a 95% confidence interval (CI). The absolute values of SMD were interpreted as follows: < 0.2 (negligible), 0.2 to < 0.5 (small), 0.5 to < 0.8 (medium), and ≥ 0.8 (large).

Between-study heterogeneity was quantified using the I^2^ statistic, interpreted as: *I*^2^ < 25% (low), 25% ≤ *I*^2^ < 50% (moderate), 50% ≤ *I*^2^ < 75% (substantial), and *I*^2^ ≥ 75% (considerable). A fixed-effect model was used if heterogeneity was not significant (*I*^2^ < 50% and *p* > 0.10); otherwise, a random-effects model was applied. Sensitivity analyses were conducted to test the robustness of the pooled results when *I*^2^ ≥ 50%. Publication bias was assessed through visual inspection of funnel plots and Egger’s regression test. If significant publication bias was indicated, the “trim and fill” method was employed to adjust the effect estimate. To ensure adequate power, publication bias analysis was performed only when at least five studies were available for an outcome.

Since the included studies used various scales to assess depression, emotion regulation, and anxietyas performed only when at least dard deviations, sample sizes). All data were entered inoint. xcluding those clearly ineligible. The full tof effect sizes. For studies where a lower score indicated improvement, the mean and standard deviation were multiplied by -1. This adjustment ensured that for all studies, a higher score consistently represented a better outcome, simplifying the interpretation of the overall analysis.

### 2.9 Subgroup analysis and meta-regression

Pre-specified subgroup analyses and meta-regressions were conducted to explore potential sources of heterogeneity and examine the influence of study-level moderators on the intervention effects, where data permitted. Subgroup analyses were performed for the outcomes of depression, anxiety, and emotion regulation based on the following *a priori* variables: (1) Exercise intensity, categorized as low (LPA, e.g., yoga, tai chi), moderate (MPA, e.g., brisk walking, jogging, typically 50–70% of maximum heart rate), or moderate-to-vigorous (MVPA, e.g., high-intensity interval training); (2) Exercise type, categorized as physical-dominant (e.g., running, swimming) or mind-body integrated (e.g., yoga, tai chi); (3) Age group, dichotomized as children ( ≤ 12 years) or adolescents ( > 12 years) based on mean age; (4) Intervention duration, categorized as a single session, ≤ 8 weeks, or > 8 weeks.

Meta-regression analysis was planned provided a sufficient number of studies (typically ≥ 10) were available to ensure statistical power (Sánchez-Meca and Botella, 2024). A random-effects meta-regression model using the restricted maximum likelihood method was employed. In this study, only the depression outcome met the minimum sample size requirement for meta-regression. Therefore, meta-regression was conducted solely for depression to explore the relationship between continuous or ordinal variables (e.g., age, intervention duration) and the effect size, with results presented as the regression coefficient (β), standard error (SE), and corresponding *p*-value.

## 3 Results

### 3.1 Literature screening and study inclusion

The literature screening and selection process followed the PRISMA statement guidelines. Systematic searches across five databasesches acro*n* = 1,352), Web of Science (*n* = 854), Embase (*n* = 780), Cochrane Library (*n* = 756), and CNKI (*n* = 79)), and CNKItotal of 3,821 initial records. After removing 1,224 duplicate records, we screened the titles and abstracts of the remaining 2,597 articles, excluding 2,127 that did not meet the inclusion criteria. Full-text articles were sought for the remaining 470 citations, of which 20 were unavailable. A detailed assessment of the 450 successfully retrieved full-text articles led to the exclusion of 428 studies. The primary reasons for exclusion were: lack of separate data for an ADHD population, unclear intervention description, no substantial difference between the intervention and control groups, absence of standardized emotional outcome measures, and insufficient statistical data. Ultimately, 22 studies met the inclusion criteria for the qualitative synthesis, and 18 of these, which fulfilled all criteria for meta-analysis, were included in the quantitative synthesis. All included studies were randomized or quasi-randomized controlled trials evaluating the effect of exercise interventions on mental health in children and adolescents with ADHD. The entire screening process was conducted independently by two researchers, with any disagreements resolved by a third researcher. The detailed flow of the literature screening process is depicted in Figure 1.

### 3.2 Characteristics of included studies and risk of bias assessment

A total of 22 empirical studies from 12 countries—namely Egypt, Israel, Canada, China, Spain, Tunisia, the United States, Australia, Germany, South Korea, Iran, and Brazil—were included in the final qualitative synthesis. Participants in all studies were diagnosed with Attention-Deficit/Hyperactivity Disorder (ADHD) according to international standards such as the Diagnostic and Statistical Manual of Mental Disorders (DSM-IV to DSM-5) or the International Classification of Diseases (ICD-10), ensuring diagnostic standardization and comparability across the sample.

The exercise interventions were diverse, primarily categorized into physical-dominant exercises (e.g., structured physical activities) and mind-body integrated exercises (e.g., yoga, tai chi). The intervention parameters varied widely: duration ranged from 1 to 20 weeks, frequency from 1 to 7 sessions per week, session length from 10 to 90 min, and intensity spanned low, moderate, and high levels, reflecting the heterogeneity of exercise prescriptions.

Control conditions included a variety of designs such as treatment-as-usual, no intervention, sedentary activities, cognitive training, and pharmacotherapy, providing effective comparators for assessing the additive effect of exercise interventions.

Outcomes were assessed using standardized and validated tools, including the Conners’ Rating Scales (CRS), Child Behavior Checklist (CBCL), and Strengths and Difficulties Questionnaire (SDQ), which quantified effects on emotional symptoms and emotion regulation, thereby ensuring the reliability of the findings (for detailed results, see Table 1).

**TABLE 1:** Characteristics of included studies.

Study	Country	Diagnostic	Experimental group						Control group		
			N	Intervention	Intensity	Duration (weeks)	Frequency (/week)	Time (min/session)	N	Control activity	Outcomes
Ahmed and Mohamed (2011)	Egypt	CRS-MV	42	Purely physical exercises	MPA	10	3	45	42	WLC	CRS
Helmer et al. (2024)	Israel	DSM-V	31	Purely physical exercises	MVPA	12	1	45	31	TAU	SDQ
Bigelow et al. (2021)	Canada	MPD	16	Mind-body exercises	MVPA	1	1	10	16	WLC	POMS-BF
Brellenthin et al. (2025)	USA	DSM-IV	20	Purely physical exercises	MVPA	12	5	45	20	TAU	CRS
García-Gómez et al. (2016)	Spain	DSM-IV-TR	9	Purely physical exercises	MPA	12	2	45	5	WLC	BASC-T
Hattabi et al. (2022)	Tunisia	DSM-IV	20	Purely physical exercises	MVPA	12	3	90	20	WLC	CBCL
Hoza et al. (2015)	USA	DSM-IV-TR	49	Purely physical exercises	MVPA	12	5	31	45	WLC	CRS
Jensen and Kenny (2004)	Australia	DSM-IV	11	Mind-body exercises	LPA	20	1	60	8	TAU	CRS
Fritz and O’Connor (2016)	USA	DSM-IV	32	Mind-body exercises	MPA	1	1	20	32	TAU	POMS-BF
Lufi and Parish-Plass (2011)	Israel	DSM-IV-TR	15	Purely physical exercises	MPA	20	1	90	17	TAU	YSRS
Meßler et al. (2018)	Germany	ICD-10	14	Mind-body exercises	MVPA	3	3	30	14	TAU	KINDL
Ontiveros et al. (2025)	South Korea	DSM-IV-TR	17	Purely physical exercises	MPA	12	2	60	17	TAU	CBCL
Pan et al. (2016)	China	DSM-IV	16	Purely physical exercises	MPA	12	2	70	16	TAU	CBCL
Sabzi et al. (2021)	Iran	MPD	20	Purely physical exercises	MVPA	8	3	30	20	TAU	CRS
Wang (2023)	China	DSM	15	Mind-body exercises	LPA	10	2	20	15	TAU	ERC
Silva et al. (2020)	Brazil	DSM-IV	10	Purely physical exercises	MPA	8	2	45	10	WLC	BAI
Li et al. (2021)	China	MPD	45	Purely physical exercises	MPA	16	2	50	45	TAU	CRS
Chen and Cheng (2016)	China	DSM-IV	18	Mind-body exercises	MPA	16	3	60	18	TAU	CBCL

CRS-MV, Conners’ Rating Scales, a parent-report measure for assessing ADHD-related symptoms in children and adolescents; MPD, Multiple Personality Disorder, a former term for Dissociative Identity Disorder; ADHD-CN, ADHD-Combined Type, an ADHD subtype characterized by both inattentive and hyperactive-impulsive symptoms; DSM, Diagnostic and Statistical Manual of Mental Disorders, a primary diagnostic standard published by the American Psychiatric Association. Diagnostic criteria may vary across editions (e.g., DSM-IV, DSM-5). DSM-IV/DSM-IV-TR, Diagnostic and Statistical Manual of Mental Disorders, Fourth Edition and Fourth Edition, Text Revision; DSM-V/DSM-5, Diagnostic and Statistical Manual of Mental Disorders, Fifth Edition (Roman numeral V and Arabic numeral 5 are used interchangeably); ICD-10, International Statistical Classification of Diseases and Related Health Problems, 10th Revision, a global diagnostic standard for all diseases and health conditions, including mental and behavioral disorders, published by the World Health Organization; CBCL, Child Behavior Checklist, a parent-report instrument for comprehensive assessment of behavioral and emotional problems in children and adolescents; BASC-T/CBRS, Behavior Assessment System for Children (Teacher/Comprehensive Version), rated by parents or teachers to evaluate behavior, emotions, and adaptive skills; CRS, Conners’ Rating Scales, a core instrument rated by parents, teachers, or self-report, specifically designed to assess ADHD symptoms; SDQ, Strengths and Difficulties Questionnaire, a brief screening tool rated by parents, teachers, or self-report for rapid assessment of behavioral and emotional problems; ERC, Emotion Regulation Checklist, rated by parents or teachers to assess a child’s emotional management capacity and stability. KINDL, Quality of Life Questionnaire for Children, a self-report measure assessing health-related quality of life from the child’s perspective; POMS-BF, Profile of Mood States, Short Form, a self-report measure for adults or adolescents assessing transient, fluctuating mood states; BDI, Beck Depression Inventory, a self-report measure for adults or adolescents assessing the severity of depressive symptoms; BAI, Beck Anxiety Inventory, a self-report measure for adults assessing the severity of anxiety symptoms; YSRS, Youth Self-Report Scale, a self-report measure for adolescents assessing competencies and behavioral problems from their own perspective.

### 3.3 Study quality and risk of bias

**FIGURE 2:**
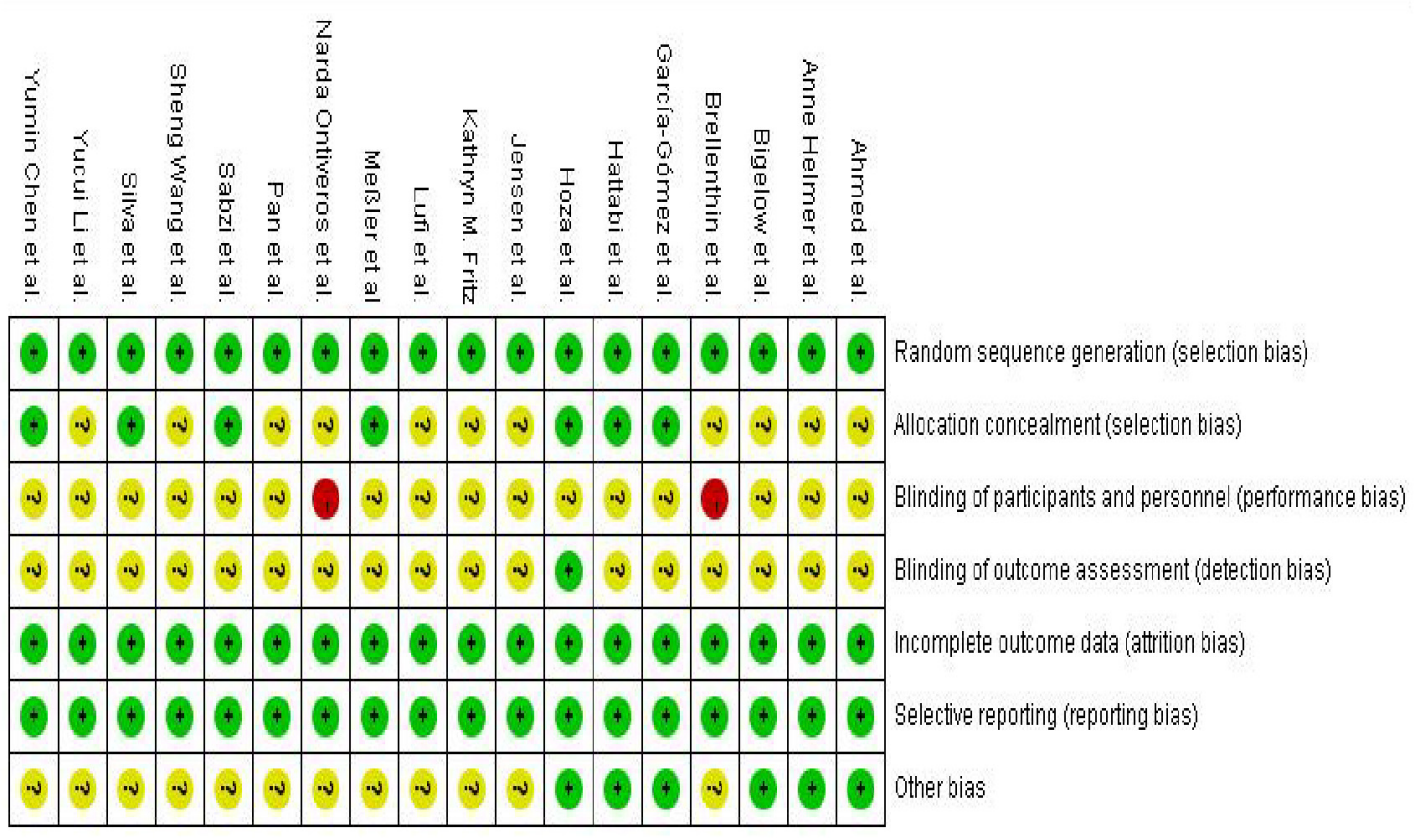
Risk of bias graph for included studies.

**FIGURE 3:**
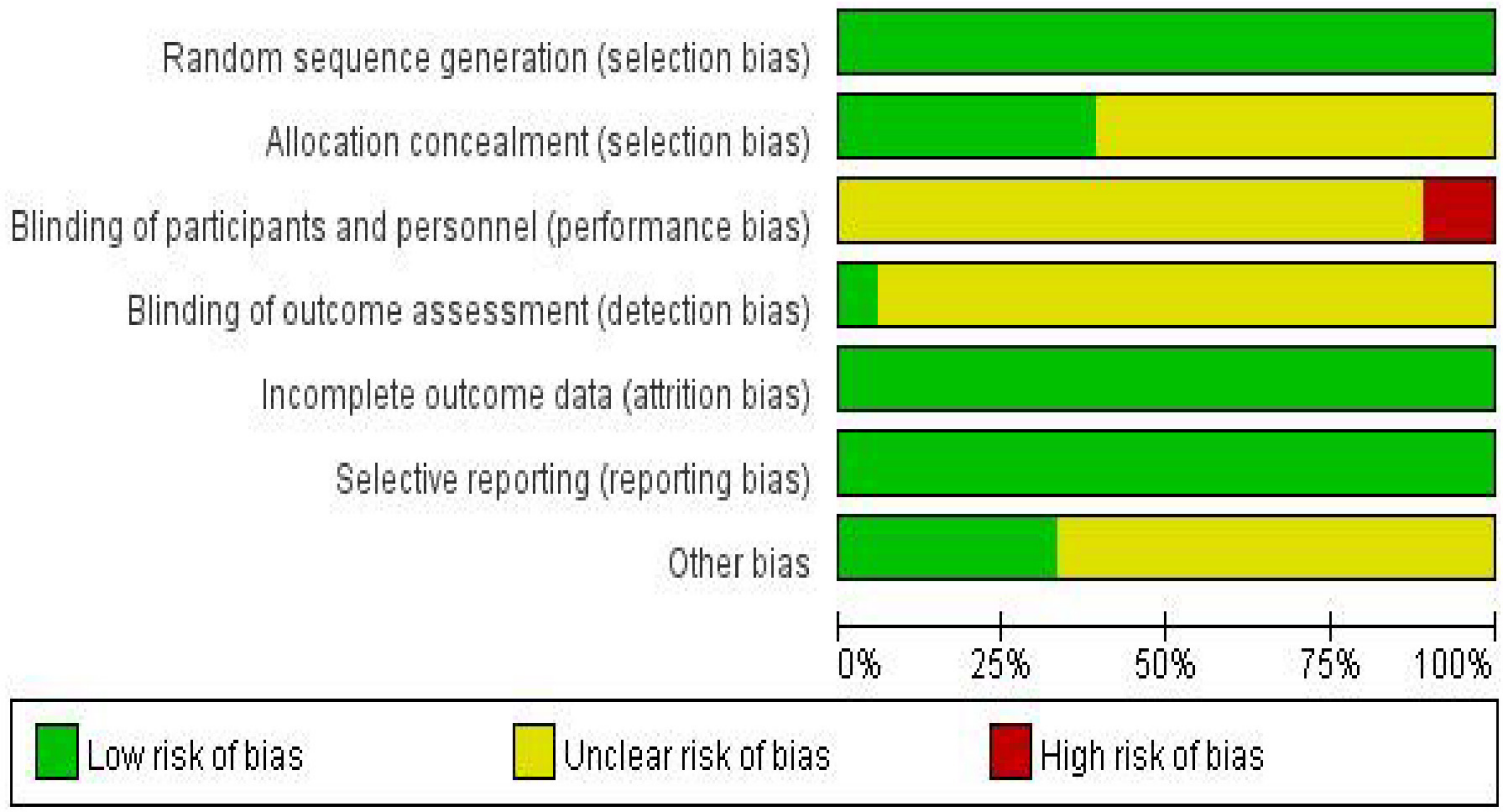
Risk of bias summary graph for included studies.

According to the pre-established protocol, we used the revised Cochrane Risk of Bias tool (ROB 2) to assess the risk of bias in the 18 included randomized controlled trials (Sterne et al., 2019). Overall, exercise intervention studies primarily faced challenges in terms of intervention adherence, implementation of blinding procedures, and allocation concealment, reflecting the inherent methodological limitations of such research. However, most studies demonstrated a low risk of bias concerning the generation of random sequences and the selective reporting of outcome data (for details, please refer to Figures 2, 3).

Simultaneously, we employed the PEDro scale to quantitatively evaluate the overall methodological quality of the included studies PEDro (2025). The PEDro scale is a widely used tool for assessing the quality of randomized controlled trials (RCTs), encompassing 11 key items related to study design, allocation methods, blinding, follow-up, and outcome reporting. Each item is scored as “yes” or “no,” with the total score reflecting the study’s overall quality. The total score ranges from 0 to 10, where a higher score indicates stronger internal validity and more reliable evidence. In this study, PEDro scores ranged from 5/10 to 8/10, indicating that the included studies generally possessed moderate to good methodological quality, although limitations such as incomplete implementation of blinding and insufficient reporting of allocation concealment information were noted (for detailed results, see Table 2).

**TABLE 2:** PEDro scale.

Included study	Eligibility criteria	Random allocation	Concealed allocation	Baseline comparability	Blinding of subjects	Blinding of therapists	Blinding of assessors	Adequate follow-up	Intention-to-Treat analysis	Between-Group comparisons	Point estimates and variability	PEDro score
Ahmed et al.	1	1	0	1	0	0	0	1	1	1	1	6/10
Anne helmer et al.	1	1	0	1	0	0	0	1	1	1	1	6/10
Bigelow et al.	1	1	0	1	0	0	0	1	1	1	1	6/10
Brellenthin et al.	1	1	0	1	0	0	0	1	0	1	1	5/10
García-Gómez et al.	1	1	0	1	0	0	0	1	1	1	1	6/10
Hattabi et al.	1	1	1	1	0	0	0	1	1	1	1	7/10
Hoza et al.	1	1	1	1	0	0	1	1	1	1	1	8/10
Jensen et al.	1	1	0	1	0	0	0	1	1	1	1	6/10
Kathryn M. fritz	1	1	0	1	0	0	0	1	1	1	1	6/10
Lufi et al.	1	1	0	1	0	0	0	1	1	1	1	6/10
Meßler et al	1	1	1	1	0	0	0	1	1	1	1	7/10
Narda ontiveros et al.	1	1	0	1	0	0	0	1	0	1	1	5/10
Pan et al.	1	1	0	1	0	0	0	1	1	1	1	6/10
Sabzi et al.	1	1	1	1	0	0	0	1	1	1	1	7/10
Sheng Wang et al.	1	1	0	1	0	0	0	1	1	1	1	6/10
Silva et al.	1	1	1	1	0	0	0	1	1	1	1	7/10
Yucui Li et al.	1	1	0	1	0	0	0	1	1	1	1	6/10
Yumin Chen et al.	1	1	0	1	0	0	0	1	1	1	1	6/10

### 3.4 Meta-analysis results

**FIGURE 4:**
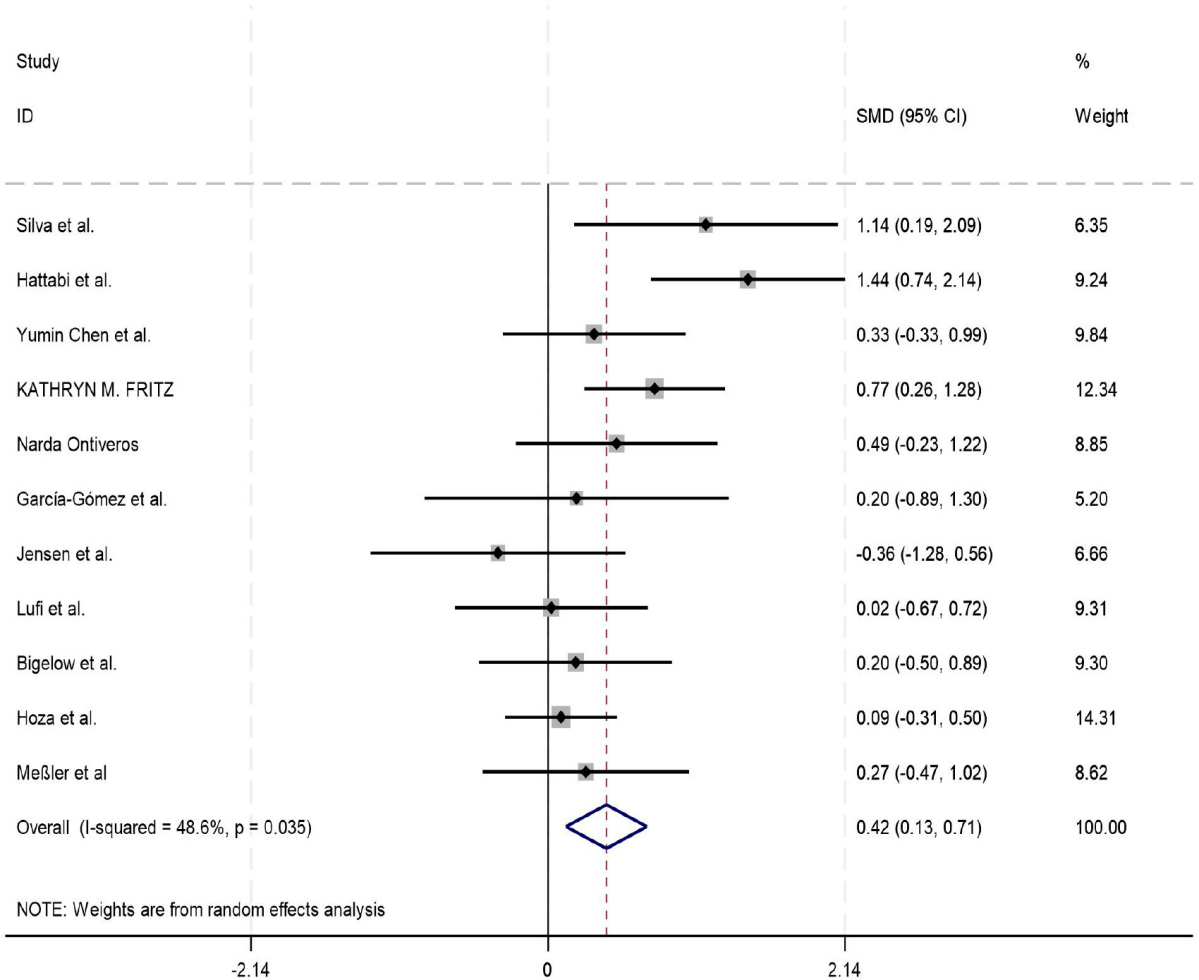
Forest plot of the effect of exercise intervention on depression in ADHD.

**FIGURE 5:**
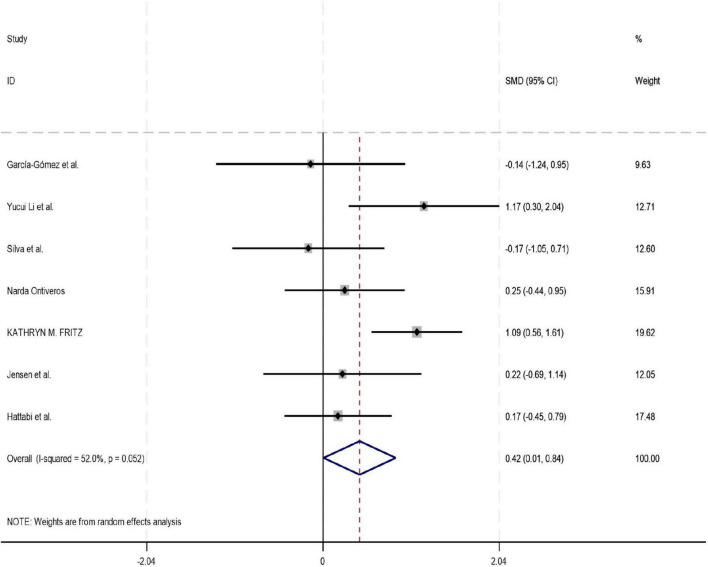
Forest plot of the effect of exercise intervention on anxiety in ADHD.

**FIGURE 6:**
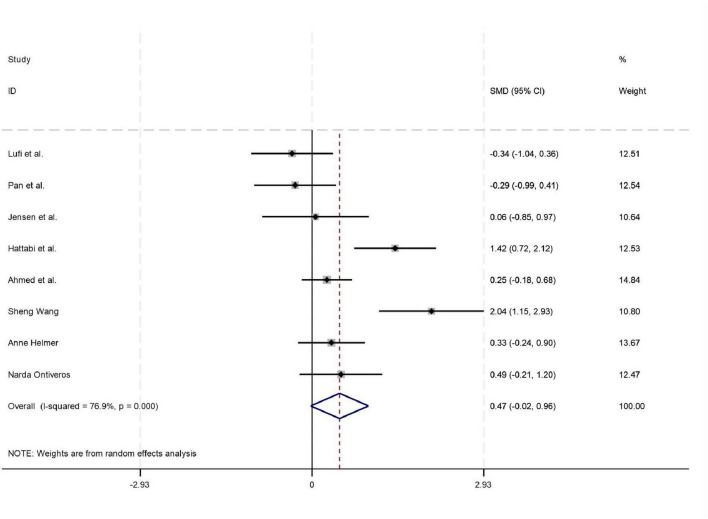
Forest plot of the effect of exercise intervention on emotion regulation in ADHD.

Among the 18 studies included in the meta-analysis, 11, 8, and 7 studies reported outcome measures for depression, emotion regulation, and anxiety, respectively. As the assessment tools varied across studies (including a total of 10 different scales such as CBCL, CRS, and BDI), the standardized mean difference (SMD) was used as the pooled effect size. Heterogeneity tests indicated the presence of heterogeneity across all outcome dimensions: depression (*I*^2^ = 48.6%), emotion regulation (*I*^2^ = 52.0%), and anxiety (*I*^2^ = 76.9%). Although the *I*^2^ value for depression was slightly below 50%, its heterogeneity test *P*-value was < 0.05. Consequently, a random-effects model was employed to pool the effect sizes for all analyses. The meta-analysis revealed that exercise intervention demonstrated significant beneficial effects on ADHD children and adolescents, showing improvement in depressive symptoms (SMD = 0.42, 95% CI: 0.129–0.712, *P* < 0.05), enhancement of emotion regulation capacity (SMD = 0.47, 95% CI: 0.06–0.924, *P* < 0.05), and reduction of anxiety symptoms (SMD = 0.43, 95% CI: 0.006–0.84, *P* < 0.05). All these differences were statistically significant (for detailed results, see Figures 4–6).

### 3.5 Subgroup and meta-regression analyses

This study systematically evaluated the influence of potential moderating variables—including exercise intensity, exercise type, age group, and intervention duration—on the effects of exercise on mental health outcomes (depression, emotion regulation, and anxiety) through subgroup analyses.

Regarding exercise intensity, subgroup analyses revealed that only moderate-intensity exercise yielded a statistically significant improvement in depressive symptoms (SMD = 0.54, 95% CI: 0.18–0.90, *P* = 0.003). The effects of low-intensity and moderate-to-vigorous intensity exercise on depression, as well as the effects of all intensity levels on emotion regulation and anxiety, were non-significant (all *P* > 0.05). In the comparison of exercise types, mind-body exercises (e.g., yoga, tai chi) demonstrated significant beneficial effects on both depression (SMD = 0.46, 95% CI: 0.15–0.78, *P* = 0.004) and anxiety (SMD = 0.80, 95% CI: 0.56–1.61, *P* = 0.002), with a particularly large effect size observed for anxiety. In contrast, purely physical exercises did not show significant effects on any of the three outcome measures (all *P* > 0.05). From the perspective of age groups, exercise interventions produced a significant improvement in depressive symptoms among adolescents (SMD = 0.35, 95% CI: 0.01–0.70, *P* = 0.046). Although a moderate effect trend was observed for children, it did not reach statistical significance (*P* = 0.059). Exercise interventions did not show significant effects on emotion regulation or anxiety in either age group. Concerning intervention duration, no statistically significant differences were found in the improvement of depression, emotion regulation, or anxiety across different intervention lengths (single session, ≤ 8 weeks, > 8 weeks; all *P* > 0.05) (for detailed results, see Table 3).

**TABLE 3:** Subgroup analysis of different moderating variables in exercise interventions on depression, emotion regulation, and anxiety.

Moderator	Subgroup category	Results of the meta-analysis on depression			Results of the meta-analysis on emotion regulation			Results of the meta-analysis on anxiety		
		SMD (95%CI)	*Z*	*P*	SMD (95%CI)	*Z*	*P*	SMD (95%CI)	*Z*	*P*
Exercise intensity	LPA	0.06(–0.60∼0.73)	0.19	0.853	1.05(–0.89∼2.99)	1.06	0.288	0.225(–0.68∼1.14)	0.59	0.554
	MPA	0.54(0.18∼0.90)	2.95	0.003	–0.02(–0.45∼0.44)	2.25	0.993	0.501(–0.067∼1.07)	0.97	0.331
	MVPA	0.48(–0.12∼1.07)	1.57	0.117	0.70(–0.67∼2.08)	5.15	0.315	0.173(–0.448∼0.794)	0.55	0.585
Type of exercise	Physical-dominant exercise	0.43(–0.04∼0.90)	1.80	0.071	0.22(–0.28∼0.71)	0.85	0.397	0.263(–0.091∼0.616)	1.46	0.145
	Mind-body integrated exercise	0.46(0.15∼0.78)	2.87	0.004	1.05(–0.89∼2.99)	1.06	0.288	1.087(0.561∼1.613)	4.05	0.002
Age group	Children	0.55(–0.02∼1.13)	1.88	0.059	0.67(–0.67∼2.01)	0.98	0.328	0.465(–0.098∼1.028)	1.62	0.106
	Adolescents	0.35(0.01∼0.70)	2.00	0.046	0.27(–0.29∼0.82)	0.94	0.347	0.333(–0.366∼1.03)	0.93	0.351
Intervention duration	a single session	0.54(–0.01∼1.08)	1.91	0.056	NA	NA	NA	NA	NA	NA
	8 weeks or less	0.65(–0.19∼1.49)	1.52	0.129	NA	NA	NA	–0.17(–1.048∼0.71)	0.38	0.705
	more than 8 weeks	0.33(–0.07∼0.74)	1.62	0.106	0.40(–0.13∼0.92)	1.49	0.137	0.336(–0.048∼0.72)	1.71	0.087

**FIGURE 7:**
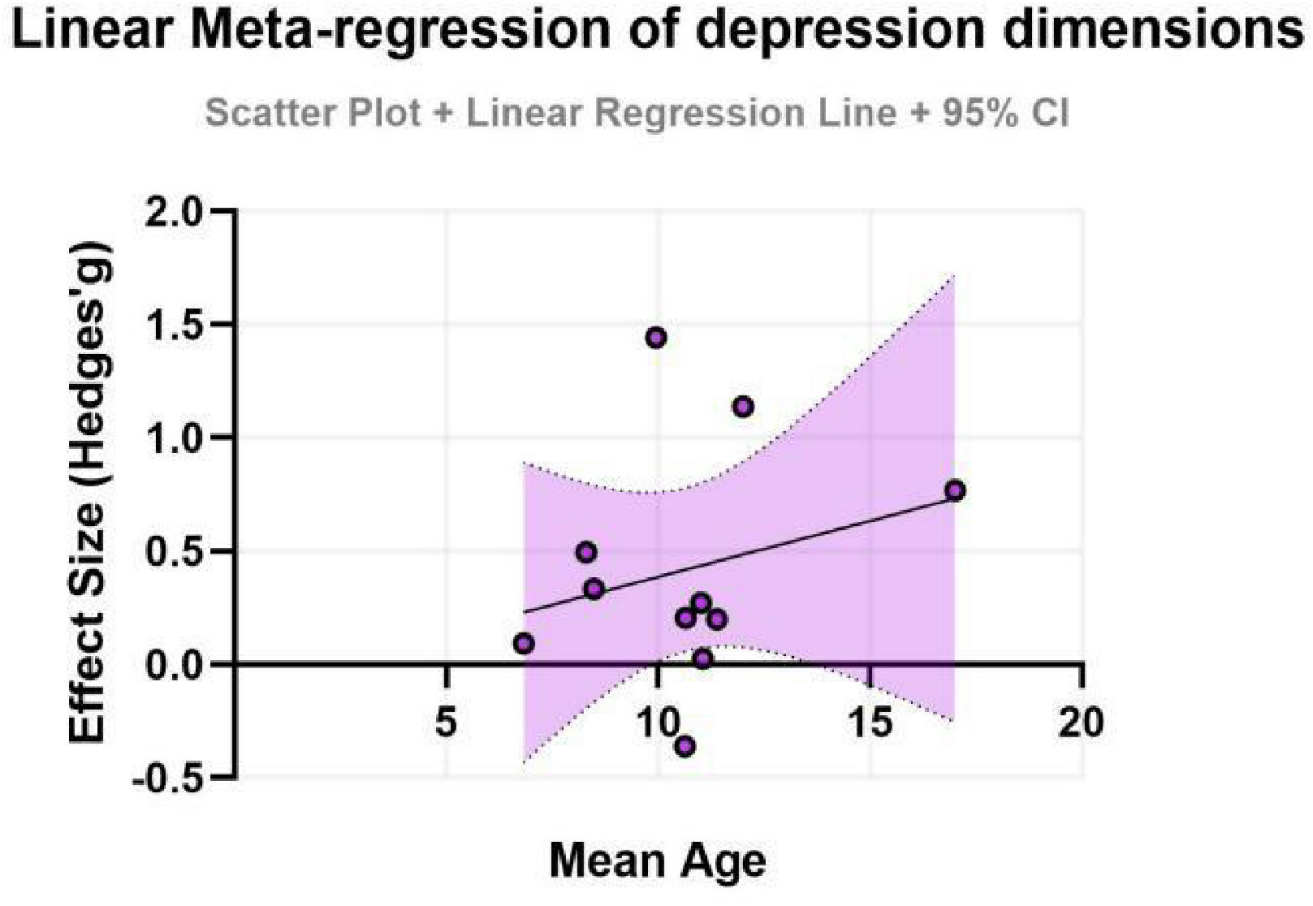
Effect sizes for depression in ADHD by age group.

**FIGURE 8:**
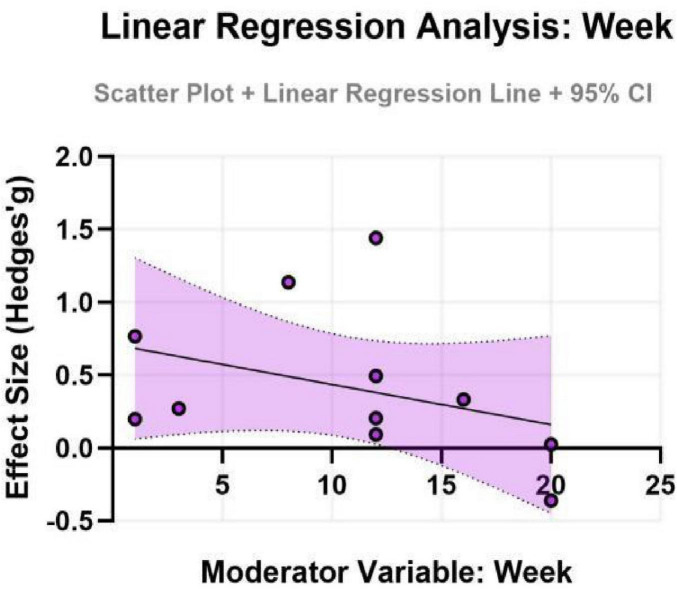
Effect sizes for depression in ADHD by intervention duration.

To further explore potential linear relationships with continuous moderators (age and intervention duration), simple meta-regression analyses were performed. The results indicated that neither age (β = -0.20, SE = 0.32, *P* = 0.552) nor intervention duration (β = -0.11, SE = 0.19, *P* = 0.564) had a statistically significant moderating effect on the improvement of depressive symptoms. These models exhibited very low explanatory power (adjusted *R*^2^ ≈ 0%), suggesting that age and duration, as single variables, cannot adequately account for the observed heterogeneity. The non-significant findings might be attributed to the relatively limited number of studies included, which constrained the statistical power of the meta-regression, or to potential complex interactions with other factors (e.g., exercise type, intensity) (for detailed results, see Figures 7, 8).

In summary, these exploratory subgroup analyses suggest that moderate-intensity and mind-body exercises might be associated with improvements in depressive and anxiety symptoms. However, the meta-regression analyses did not detect significant linear moderating effects for age or intervention duration as continuous variables. Given the limited number of studies within certain subgroups and the substantial heterogeneity observed, these findings should be interpreted with caution and viewed as hypothesis-generating rather than definitive. The heterogeneity among studies may stem from other unmeasured factors, complex interactions among multiple variables, or the inherent limitations of the current sample size. These preliminary insights may inform the design of future research, but more robust evidence is needed before definitive clinical prescriptions can be made.

### 3.6 Publication bias

To assess the potential for publication bias in this meta-analysis, we performed both a visual inspection of the funnel plot asymmetry (Peters et al., 2008) and Egger’s linear regression test (Irwig et al., 1998) to evaluate publication bias statistically (Afonso et al., 2024).

**FIGURE 9:**
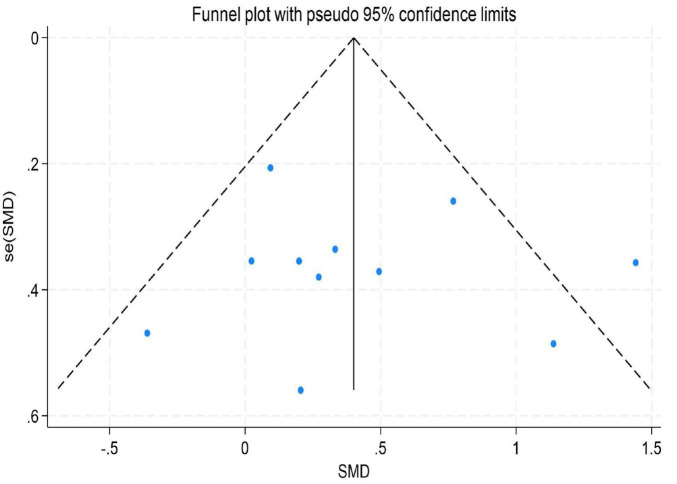
Funnel plot for depression.

**FIGURE 10:**
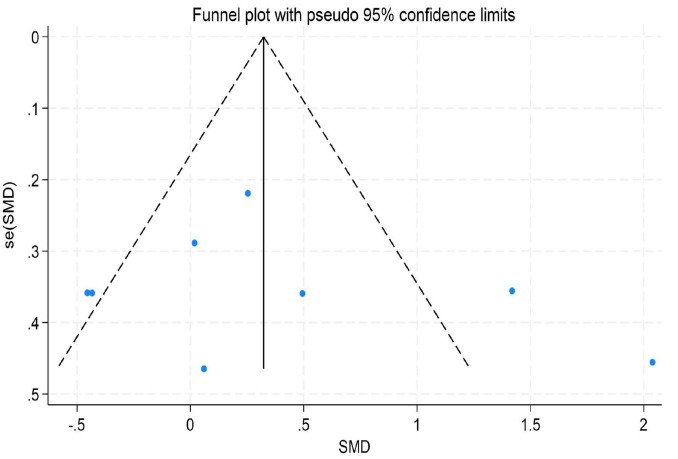
Funnel plot for emotion regulation.

**FIGURE 11:**
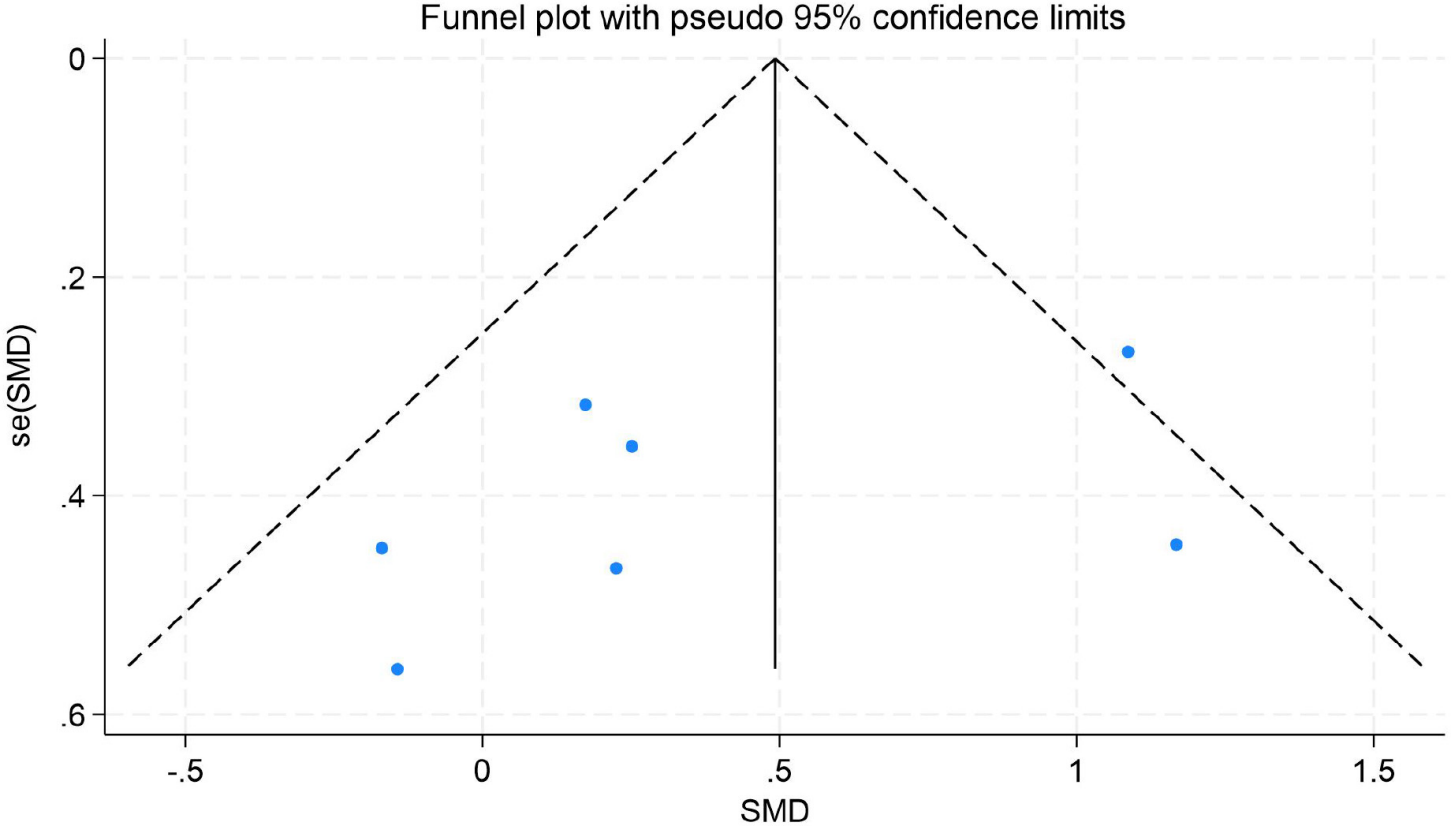
Funnel plot for anxiety.

The results indicated no significant evidence of publication bias. For depression, Egger’s test yielded a *z*-value of 0.39 (*P* = 0.742 > 0.05), suggesting that the funnel plot was largely symmetrical. For emotion regulation, the test resulted in a *z*-value of 0.49 (*P* = 0.504 > 0.05), also indicating good symmetry of the funnel plot. Similarly, for anxiety, the test produced a *z*-value of 1.05 (*P* = 0.230 > 0.05), demonstrating no significant asymmetry. These findings imply that the distribution of effect sizes from the included studies was relatively even for depression, emotion regulation, and anxiety. There was no systematic bias indicative of the preferential publication of studies with statistically significant results or larger effect sizes. Consequently, these results enhance the credibility and external validity of the findings from this meta-analysis (for detailed results, see Figures 9–11).

## 4 Discussion

This meta-analysis comprehensively evaluated the efficacy of exercise interventions on the mental health of children and adolescents with Attention-Deficit/Hyperactivity Disorder (ADHD), specifically targeting depressive symptoms, anxiety symptoms, and emotion regulation ability. It further explored the moderating effects of key parameters such as exercise type, intensity, duration, and participant age group. The primary findings include: (1) Exercise interventions had significant positive effects on improving depressive symptoms, anxiety symptoms, and emotion regulation ability in individuals with ADHD; (2) The psychological benefits of exercise were significantly moderated by exercise type and intensity, with mind-body exercises and moderate-intensity exercise showing particularly prominent effects on depressive symptoms; (3) Meta-regression analyses suggested potential age-related differences in intervention effects, although the statistical robustness of some moderating effects requires further validation due to the limited number of included studies. The following sections provide an in-depth interpretation of these findings.

### 4.1 Overall benefits of exercise intervention on mental health in ADHD

The pooled results of this meta-analysis indicate that exercise interventions significantly improved depressive symptoms (SMD = 0.42, 95% CI: 0.13–0.71) and emotion regulation ability (SMD = 0.47, 95% CI: 0.06–0.92) in individuals with ADHD, and showed a positive trend toward alleviating anxiety symptoms (SMD = 0.43, 95% CI: 0.006–0.84). This finding provides high-level evidence supporting the establishment of exercise intervention as a valuable non-pharmacological adjunctive approach within the comprehensive management of ADHD. The mental health benefits derived from exercise may stem from multi-level physiological and psychological mechanisms. Physiologically, aerobic exercise promotes the release of Brain-Derived Neurotrophic Factor (BDNF) and enhances neuroplasticity in the prefrontal cortex and limbic system (e.g., hippocampus, amygdala), which collectively regulate emotional processing and stress responses (Kandola et al., 2019). Concurrently, exercise modulates autonomic nervous system function, increasing parasympathetic tone, thereby reducing physiological arousal levels and alleviating emotional dysregulation and anxiety commonly seen in ADHD (Chang et al., 2012). From a psycho-behavioral perspective, exercise provides a predictable environment, clear rules, and immediate feedback for individuals with ADHD, which helps enhance their self-efficacy and sense of control over emotions (Xie et al., 2021). Social interactions within group-based sports also offer valuable opportunities for learning emotional expression and regulation skills (Xie et al., 2021).

### 4.2 Specific effects of exercise type and intensity

Subgroup analyses revealed significant moderating effects of exercise prescription parameters on psychological benefits, providing critical insights for developing precise intervention protocols. Firstly, regarding exercise type, mind-body exercises (e.g., yoga, tai chi) demonstrated significant medium-to-large effect sizes for both depression (SMD = 0.46, 95% CI: 0.15–0.78, *P* = 0.004) and anxiety (SMD = 0.80, 95% CI: 0.56–1.61, *P* = 0.002), outperforming purely physical exercises (Li et al., 2023). This advantage may be attributed to the unique “mind-body integration” characteristic of these activities: they involve physical exertion while emphasizing awareness of breath, focused attention, and acceptance of present-moment physical and mental states (Li et al., 2023). This mode of practice directly trains core components of attention and emotion regulation—response inhibition and metacognitive monitoring—thus more directly targeting the pathophysiological core of emotional dysregulation in ADHD (Barkley and Fischer, 2010). Secondly, concerning exercise intensity, moderate-intensity exercise yielded the most significant improvement in depressive symptoms (SMD = 0.54, 95% CI: 0.18–0.90, *P* = 0.003), whereas the effects of low-intensity and high-intensity exercises were non-significant. This finding aligns with the “inverted-U” dose-response hypothesis (McMorris and Hale, 2012), which posits that moderate physiological stress optimally activates neuroendocrine systems (e.g., promoting BDNF secretion, modulating cortisol levels). In contrast, intensities that are too low may fail to induce sufficient neural adaptation, while those too high might lead to excessive fatigue and negative emotional experiences, thereby attenuating the intervention effect (Heijnen et al., 2016).

### 4.3 Potential moderating roles of age and intervention duration

Subgroup analyses indicated that the exercise intervention significantly improved depressive symptoms in adolescents (SMD = 0.35, 95% CI: 0.01–0.70, *P* = 0.046), whereas the effect for children, although showing a moderate trend, did not reach statistical significance. This discrepancy may reflect differences in neuroplasticity and psychosocial needs across developmental stages. Adolescence is a period of high incidence for depressive symptoms, and cognitive abilities at this stage are sufficiently developed to better comprehend and benefit from the psychological advantages of exercise (Kessler et al., 2007). For children, exercise interventions might need to place greater emphasis on fun and gamification; the sensitivity of emotional outcome measures in this group might be lower compared to behavioral measures, which could partly explain the non-significant result (Neudecker et al., 2019). The negative coefficient for age in the meta-regression (albeit non-significant) also suggests that for older individuals with ADHD, intervention strategies might need adjustment, such as incorporating cognitive challenges or psychoeducation, to maintain efficacy. Furthermore, the role of intervention duration appears complex. Single sessions demonstrated immediate anxiety-reducing effects, while sustained, long-term interventions seem crucial for consolidating psychological benefits (Ashor et al., 2015). Future research, through well-designed trials, is needed to clarify the optimal duration and frequency for different age groups.

### 4.4 Physiological mechanisms of exercise-induced improvement in mental health for ADHD

The positive effects of exercise on depression, anxiety, and emotion regulation in ADHD are underpinned by synergistic physiological changes across multiple systems. Neurochemically, exercise upregulates monoaminergic neurotransmitters (e.g., serotonin, norepinephrine, dopamine) in key emotion-regulation regions such as the prefrontal cortex, hippocampus, and amygdala. This directly improves neural transmission linked to mood enhancement (Dey et al., 1992). Concurrently, exercise-induced increases in BDNF expression promote neuronal survival and synaptic plasticity, counteracting stress-induced atrophy and providing a structural basis for long-term antidepressant effects (Alizadeh and Dehghanizade, 2022; Coll-Padrós et al., 2019). At the neural network level, evidence suggests exercise normalizes hyperactivity and aberrant connectivity in the Default Mode Network (DMN) and Salience Network (SN), which are often dysfunctional in ADHD (Mehren et al., 2019). This optimization constitutes the neural foundation for effective emotion regulation. Finally, exercise modulates autonomic function by increasing vagal tone and heart rate variability, thereby improving physiological hyper-reactivity to emotional stress (Cortese et al., 2012; Liang et al., 2019). Collectively, these multi-level adaptations—spanning neurotransmitter balance, neurotrophic support, network integration, and autonomic regulation—constitute the biological mechanisms through which exercise alleviates comorbid symptoms and enhances emotion regulation in ADHD (Lin and Kuo, 2013; Ontiveros et al., 2025).

### 4.5 Clinical implications and implementation recommendations

The findings of this study provide evidence-based support for integrating exercise interventions into the clinical management of ADHD; however, these recommendations must be framed with caution considering the variability in intervention protocols and study quality observed in the existing literature. Specifically, while moderate-intensity aerobic exercise and mind-body exercises show promise for alleviating depression and anxiety, the heterogeneity in intensity measurement and duration across studies suggests that individual responses may vary. Therefore, exercise prescriptions should be personalized and closely monitored, acknowledging the current lack of standardized “dose-response” guidelines (Lin, 2021).

Crucially, it is essential to emphasize that exercise should be considered a complementary (adjunctive) intervention rather than a stand-alone treatment for mental health outcomes in ADHD. Given the complexity of ADHD and its comorbidities, exercise is most effective when integrated into a multimodal treatment plan that includes pharmacotherapy, behavioral therapy, or psychoeducation as indicated. Clinicians should avoid overgeneralizing these findings and instead view exercise as a valuable, evidence-based component of a broader, holistic management strategy aimed at improving the overall quality of life for individuals with ADHD. Future clinical implementation should involve interdisciplinary collaboration to tailor interventions to the developmental stage and symptom profile of the patient, while maintaining realistic expectations regarding the magnitude of psychological benefits.

### 4.6 Limitations and future research directions

Although this meta-analysis provides integrated evidence supporting exercise interventions for improving mental health in individuals with ADHD, several limitations warrant careful consideration when interpreting the conclusions, and these limitations also chart a course for future research (Mayer et al., 2025).

Firstly, on methodological grounds, the overall number of included studies and their sample sizes remain relatively limited. This may have constrained the statistical power in subgroup analyses, particularly in the meta-regression, to detect the true effects of moderating variables. Some subgroups (e.g., the effect of different intervention durations on emotion regulation) could not be analyzed in depth due to an insufficient number of studies. Future research needs to include more high-quality, large-sample randomized controlled trials to enhance the robustness of the evidence (Mayer et al., 2025).

Secondly, significant heterogeneity exists in the parameters of the exercise interventions across the available literature. For instance, exercise intensity was often based on subjective descriptions rather than objective, quantifiable measures like heart rate monitoring, and intervention duration and frequency varied widely. This inconsistency may confound the precise interpretation of the optimal “dose-response” relationship. Therefore, future studies should employ more standardized exercise prescriptions and objective quantification tools to clarify the optimal protocols (regarding type, intensity, frequency, and duration) for specific mental health outcomes.

Thirdly, the robustness of the pooled effect sizes is influenced by residual heterogeneity and the risk of bias. Despite conducting subgroup analyses, substantial unexplained heterogeneity remained in several outcomes, indicating that other unmeasured factors—such as instructor expertise, baseline symptom severity, or specific environmental contexts—may significantly influence intervention effects. This residual variability suggests that the pooled estimates represent an average effect that may not apply uniformly to all settings. Additionally, most included studies were rated as having a “moderate” risk of bias, largely due to the inherent challenge of blinding participants and personnel in non-pharmacological exercise trials. The lack of blinding can introduce performance and detection biases, potentially inflating the observed effect sizes. Consequently, while the overall trends are promising, the magnitude of the benefits reported here should be interpreted with these methodological constraints in mind.

## 5 Conclusion

In summary, this meta-analysis, by systematically synthesizing existing empirical evidence, clearly demonstrates that exercise intervention, as a non-pharmacological adjunctive approach, has a significant and positive effect on improving depressive and anxiety symptoms, as well as emotion regulation ability, in individuals with ADHD, with effect sizes ranging from small to moderate. More importantly, subgroup analyses further revealed that moderate-intensity and mind-body exercises may yield relatively superior benefits for alleviating depressive symptoms, while the intervention effects exhibited distinct patterns across different age groups (e.g., adolescents) and intervention durations. These findings not only establish the value of exercise for mental health in ADHD at the behavioral level but also suggest that the underlying mechanisms likely involve multi-level, integrated physiological processes, ranging from neurochemical balance and functional optimization of brain networks to autonomic nervous system regulation. Despite limitations such as heterogeneity among the included studies and a lack of direct physiological evidence, the present findings provide crucial evidence-based justification for clinical practice, underscoring the necessity and feasibility of incorporating individualized and structured exercise prescriptions into comprehensive ADHD management plans. Future research should focus on employing more rigorous designs, objective measurement indicators, and in-depth exploration of individual difference factors to further elucidate the mechanisms of action, thereby guiding the development of precise and effective exercise intervention strategies tailored to the characteristics of individuals with ADHD.

## Data Availability

The original contributions presented in the study are included in the article/supplementary material, further inquiries can be directed to the corresponding author.

## References

[B1] Afonso J. Ramirez-Campillo R. Clemente F. M. Büttner F. C. Andrade R. (2024). The perils of misinterpreting and misusing “Publication Bias” in meta-analyses: An education review on funnel plot-based methods. *Sports Med.* 54 257–269. 10.1007/s40279-023-01927-9 37684502 PMC10933152

[B2] Ahmed G. M. Mohamed S. (2011). Effect of regular aerobic exercises on behavioral, cognitive and psychological response in patients with attention deficit-hyperactivity disorder. *Life Sci. J.* 8 366–371.

[B3] Alizadeh M. Dehghanizade J. (2022). The effect of functional training on level of brain-derived neurotrophic factor and functional performance in women with obesity. *Physiol. Behav.* 251:113798. 10.1016/j.physbeh.2022.113798 35378105

[B4] Amir-Behghadami M. Janati A. (2020). Population, Intervention, Comparison, Outcomes and Study (PICOS) design as a framework to formulate eligibility criteria in systematic reviews. *Emerg. Med. J.* 37:387. 10.1136/emermed-2020-209567 32253195

[B5] Ashor A. W. Lara J. Siervo M. Celis-Morales C. Oggioni C. Jakovljevic D. G. (2015). Exercise modalities and endothelial function: A systematic review and dose–response meta-analysis of randomized controlled trials. *Sports Med.* 45 279–296. 10.1007/s40279-014-0272-9 25281334

[B6] Barkley R. A. Fischer M. (2010). The unique contribution of emotional impulsiveness to impairment in major life activities in hyperactive children as adults. *J. Am. Acad. Child Adolesc. Psychiatry* 49 503–513. 10.1097/00004583-201005000-00011 20431470

[B7] Biddle S. J. H. Asare M. (2011). Physical activity and mental health in children and adolescents: A review of reviews. *Br. J. Sports Med.* 45 886–895. 10.1136/bjsports-2011-090185 21807669

[B8] Bigelow H. Gottlieb M. D. Ogrodnik M. Graham J. D. Fenesi B. (2021). The differential impact of acute exercise and mindfulness meditation on executive functioning and psycho-emotional well-being in children and youth with ADHD. *Front. Psychol.* 12:660845. 10.3389/fpsyg.2021.660845 34194365 PMC8236645

[B9] Brellenthin A. G. Brown D. R. Connor P. J. (2025). Up for debate: Does regular physical activity really improve mental health? *Med. Sci. Sports Exerc.* 57 1056–1066. 10.1249/MSS.0000000000003636 39696764

[B10] Caldwell D. M. Welton N. J. (2016). Approaches for synthesising complex mental health interventions in meta-analysis. *Evid. Based Ment. Health* 19 16–21. 10.1136/eb-2015-102275 26792834 PMC10699336

[B11] Chang Y. J. Liu X. M. Yan Z. K. (2015). A clinical study on related factors of attention deficit hyperactivity disorder and the therapeutic effect of Yigan San. *Zhongguo Linchuang Yaolixue Zazhi* 31 1480–1483. 10.13699/j.cnki.1001-6821.2015.15.004

[B12] Chang Y. Liu S. Yu H. Lee Y. (2012). Effect of acute exercise on executive function in children with attention deficit hyperactivity disorder. *Arch. Clin. Neuropsychol.* 27 225–237. 10.1093/arclin/acr094 22306962

[B13] Chen Y. M. Cheng L. (2016). Effects of Tai Chi exercise on children with tendencies of attention deficit and hyperactivity disorder. *J. Chengdu Sport Univ.* 42 29–32. 10.15942/j.jcsu.2016.05.005

[B14] Coll-Padrós N. León M. Valech N. Ros E. Vidal J. Estruch R. (2019). Physical activity is associated with better global cognition and frontal function in overweight/obese older adults with metabolic syndrome. *Eur. Rev. Aging Phys. Act.* 16:23. 10.1186/s11556-019-0229-y 31867067 PMC6898945

[B15] Cortese S. Kelly C. Chabernaud C. Proal E. Di Martino A. Milham M. P. (2012). Toward systems neuroscience of ADHD: A meta-analysis of 55 fMRI studies. *Am. J. Psychiatry* 169 1038–1055. 10.1176/appi.ajp.2012.11101521 22983386 PMC3879048

[B16] Dahiya A. V. Breaux R. Pham S. N. Martino D. C. Fok M. Albright J. (2025). Using a mobile app to support parents of children with behavior problems. *Res. Child Adolesc. Psychopathol.* 53 1879–1892. 10.1007/s10802-025-01385-z 41166028 PMC12718277

[B17] de la Pena I. C. Pan M. C. Thai C. G. Alisso T. (2020). Attention-deficit/hyperactivity disorder predominantly inattentive subtype/presentation: Research progress and translational studies. *Brain Sci.* 10:292. 10.3390/brainsci10050292 32422912 PMC7287898

[B18] Dey S. Singh R. H. Dey P. K. (1992). Exercise training: Significance of regional alterations in serotonin metabolism of rat brain in relation to antidepressant effect of exercise. *Physiol. Behav.* 52 1095–1099. 10.1016/0031-9384(92)90465-E 1283013

[B19] Drevon D. Fursa S. R. Malcolm A. L. (2017). Intercoder reliability and validity of webplotdigitizer in extracting graphed data. *Behav. Modif.* 41 323–339. 10.1177/0145445516673998 27760807

[B20] Eime R. M. Young J. A. Harvey J. T. Charity M. J. Payne W. R. (2013). A systematic review of the psychological and social benefits of participation in sport for children and adolescents: Informing development of a conceptual model of health through sport. *Int. J. Behav. Nutr. Phys. Act.* 10:98. 10.1186/1479-5868-10-98 23945179 PMC3751802

[B21] Faraone S. V. Glatt S. J. (2010). A comparison of the efficacy of medications for adult attention-deficit/hyperactivity disorder using meta-analysis of effect sizes. *J. Clin. Psychiatry* 71 754–763. 10.4088/JCP.08m04902pur 20051220

[B22] Faraone S. V. Biederman J. Spencer T. J. Aleardi M. (2006). Comparing the efficacy of medications for ADHD using meta-analysis. *MedGenMed* 8:4. PMC1868385 17415287

[B23] Fietsam A. C. Tucker J. R. Kamath M. S. Huang-Pollock C. Wang Z. Neely K. A. (2022). Manual dexterity and strength and in young adults with and without Attention-Deficit/Hyperactivity Disorder (ADHD). *Neurosci. Lett.* 766:136349. 10.1016/j.neulet.2021.136349 34785312 PMC9578534

[B24] Follmann D. Elliott P. Suh I. Cutler J. (1992). Variance imputation for overviews of clinical trials with continuous response. *J. Clin. Epidemiol.* 45 769–773. 10.1016/0895-4356(92)90054-q 1619456

[B25] Fritz K. M. O’Connor P. J. (2016). Acute exercise improves mood and motivation in young men with ADHD symptoms. *Med. Sci. Sports Exerc.* 48 1153–1160. 10.1249/MSS.0000000000000864 26741120

[B26] García-Gómez A. Rodríguez-Jiménez M. Guerrero-Barona E. Rubio-Jiménez J. C. García-Peña I. Moreno-Manso J. M. (2016). Benefits of an experimental program of equestrian therapy for children with ADHD. *Res. Dev. Disabil.* 59 176–185. 10.1016/j.ridd.2016.09.003 27614276

[B27] Hattabi S. Forte P. Kukic F. Bouden A. Have M. Chtourou H. (2022). A Randomized trial of a swimming-based alternative treatment for children with attention deficit hyperactivity disorder. *Int. J. Environ. Res. Public Health* 19:16238. 10.3390/ijerph192316238 36498313 PMC9739874

[B28] Heijnen S. Hommel B. Kibele A. Colzato L. S. (2016). Neuromodulation of aerobic exercise-a review. *Front. Psychol.* 6:1890. 10.3389/fpsyg.2015.01890 26779053 PMC4703784

[B29] Helmer A. Delore E. Bart O. (2024). Emotional and motor improvements in children with ADHD following equine-assisted occupational therapy. *OTJR* 46 23–32. 10.1177/15394492241307843 39716988 PMC12640361

[B30] Hoza B. Smith A. L. Shoulberg E. K. Linnea K. S. Dorsch T. E. Blazo J. A. (2015). A randomized trial examining the effects of aerobic physical activity on attention-deficit/hyperactivity disorder symptoms in young children. *J. Abnorm. Child Psychol.* 43 655–667. 10.1007/s10802-014-9929-y 25201345 PMC4826563

[B31] Irwig L. Macaskill P. Berry G. Glasziou P. (1998). Bias in meta-analysis detected by a simple, graphical test. Graphical test is itself biased. *BMJ* 316 470–471. 10.1136/bmj.315.7109.629 9492687 PMC2665595

[B32] Jensen P. S. Kenny D. T. (2004). The effects of yoga on the attention and behavior of boys with Attention-Deficit/hyperactivity Disorder (ADHD). *J. Atten. Disord.* 7 205–216. 10.1177/108705470400700403 15487477

[B33] Kandola A. Ashdown-Franks G. Hendrikse J. Sabiston C. M. Stubbs B. (2019). Physical activity and depression: Towards understanding the antidepressant mechanisms of physical activity. *Neurosci. Biobehav. Rev.* 107 525–539. 10.1016/j.neubiorev.2019.09.040 31586447

[B34] Kessler R. C. Amminger G. P. Aguilar-Gaxiola S. Alonso J. Lee S. Ustün T. B. (2007). Age of onset of mental disorders: A review of recent literature. *Curr. Opin. Psychiatry* 20 359–364. 10.1097/YCO.0b013e32816ebc8c 17551351 PMC1925038

[B35] Kouros I. Isaksson M. Ekselius L. Ramklint M. (2024). A cluster analysis of attachment styles in patients with borderline personality disorder, bipolar disorder and ADHD. *Borderline Personal. Disord. Emot. Dysregul.* 11:26. 10.1186/s40479-024-00271-2 39472982 PMC11523661

[B36] Kriel Y. Askew C. D. Solomon C. (2018). The effect of running versus cycling high-intensity intermittent exercise on local tissue oxygenation and perceived enjoyment in 18-30-year-old sedentary men. *PeerJ* 6:e5026. 10.7717/peerj.5026 29942693 PMC6014319

[B37] Li X. L. Liu X. D. Sun X. M. Wu X. B. Sang Y. L. Xue H. (2023). Effects of the modified traditional exercise “Seven-Character Concentration Exercise” on attention and sensory integration in children with attention deficit hyperactivity disorder. *J. Math. Med.* 36 601–608. 10.12173/j.issn.1004-4337.202303183

[B38] Li Y. C. Wang S. Li D. N. (2021). Observation on the effect of aerobic exercise intervention training led by team games and music rhythm on school-age children with attention deficit hyperactivity disorder. *J. Shanxi Health Vocat. Coll.* 31 122–123. 10.20281/j.cnki.wjyxb.2021.05.076

[B39] Liang X. Tang J. Chao F. Zhang Y. Chen L. Wang F. (2019). Exercise improves depressive symptoms by increasing the number of excitatory synapses in the hippocampus of CUS-Induced depression model rats. *Behav. Brain Res.* 374:112115. 10.1016/j.bbr.2019.112115 31369775

[B40] Lin F. G. (2021). Observation on the curative effect of suspension training in children with attention deficit hyperactivity disorder. *Massage Rehabil. Med.* 12 57–59. 10.19787/j.issn.1008-1879.2021.20.020

[B41] Lin T. Kuo Y. (2013). Exercise benefits brain function: The monoamine connection. *Brain Sci.* 3 39–53. 10.3390/brainsci3010039 24961306 PMC4061837

[B42] Liu C. Yang Y. Wong S. H. Leung A. Sit C. H. (2025). The effects of physical activity on mental health in adolescents with attention-deficit hyperactivity disorder: A randomized controlled trial. *Int. J. Behav. Nutr. Phys. Act.* 22:47. 10.1186/s12966-025-01745-4 40247314 PMC12007287

[B43] Lufi D. Parish-Plass J. (2011). Sport-based group therapy program for boys with ADHD or with other behavioral disorders. *Child Fam. Behav. Ther.* 33 217–230. 10.1080/07317107.2011.596000

[B44] Martz E. Weiner L. Weibel S. (2023). Identifying different patterns of emotion dysregulation in adult ADHD. *Borderline Personal. Disord. Emot. Dysregul.* 10:28. 10.1186/s40479-023-00235-y 37743484 PMC10519076

[B45] Mayer J. S. Kohlhas L. Stermann J. Medda J. Brandt G. A. Grimm O. (2025). Bright light therapy versus physical exercise to prevent co-occurring depression in adolescents and young adults with attention-deficit/hyperactivity disorder: A multicentre, three-arm, randomised controlled, pilot phase-IIa trial. *Eur. Arch. Psychiatry Clin. Neurosci.* 275 653–665. 10.1007/s00406-024-01784-1 38627266 PMC11946981

[B46] McMorris T. Hale B. J. (2012). Differential effects of differing intensities of acute exercise on speed and accuracy of cognition: A meta-analytical investigation. *Brain Cogn.* 80 338–351. 10.1016/j.bandc.2012.09.001 23064033

[B47] Mechler K. Banaschewski T. Hohmann S. Häge A. (2022). Evidence-based pharmacological treatment options for ADHD in children and adolescents. *Pharmacol. Ther.* 230:107940. 10.1016/j.pharmthera.2021.107940 34174276

[B48] Mehren A. Özyurt J. Lam A. P. Brandes M. Müller H. H. O. Thiel C. M. (2019). Acute effects of aerobic exercise on executive function and attention in adult patients with ADHD. *Front. Psychiatry* 10:132. 10.3389/fpsyt.2019.00132 30971959 PMC6443849

[B49] MeSH (2025). *Download MeSH Data.* Available online at: https://www.nlm.nih.gov/databases/download/mesh.html (accessed November 3, 2025).

[B50] Meßler C. F. Holmberg H. Sperlich B. (2018). Multimodal therapy involving high-intensity interval training improves the physical fitness, motor skills, social behavior, and quality of life of boys with ADHD: A randomized controlled study. *J. Atten. Disord.* 22 806–812. 10.1177/1087054716636936 27013028

[B51] Murray A. L. Wong S. Obsuth I. Rhodes S. Eisner M. Ribeaud D. (2021). An ecological momentary assessment study of the role of emotional dysregulation in co-occurring ADHD and internalising symptoms in adulthood. *J. Affect. Disord.* 281 708–713. 10.1016/j.jad.2020.11.086 33234281

[B52] Neudecker C. Mewes N. Reimers A. K. Woll A. (2019). Exercise interventions in children and adolescents with ADHD: A systematic review. *J. Atten. Disord.* 23 307–324. 10.1177/1087054715584053 25964449

[B53] Ontiveros N. Wiklund C. A. Ohlis A. Ekblom Ö. (2025). The role of physical activity in the association between ADHD and emotional dysregulation. *J. Affect. Disord.* 376 68–75. 10.1016/j.jad.2025.01.127 39889934

[B54] Page M. J. McKenzie J. E. Bossuyt P. M. Boutron I. Hoffmann T. C. Mulrow C. D. (2021). The PRISMA 2020 statement: An updated guideline for reporting systematic reviews. *BMJ* 372:n71. 10.1136/bmj.n71 33782057 PMC8005924

[B55] Pan C. Chu C. Tsai C. Lo S. Cheng Y. Liu Y. (2016). A racket-sport intervention improves behavioral and cognitive performance in children with attention-deficit/hyperactivity disorder. *Res. Dev. Disabil.* 57 1–10. 10.1016/j.ridd.2016.06.009 27344348

[B56] PEDro (2025). *PEDro Scale. PEDro.* Available online at: https://pedro.org.au/english/resources/pedro-scale/ (accessed November 3, 2025).

[B57] Peters J. L. Sutton A. J. Jones D. R. Abrams K. R. Rushton L. (2008). Contour-enhanced meta-analysis funnel plots help distinguish publication bias from other causes of asymmetry. *J. Clin. Epidemiol.* 61 991–996. 10.1016/j.jclinepi.2007.11.010 18538991

[B58] Sabzi A. Dana A. Salehian M. H. Yekta S. (2021). The effect of water treadmill exercise on children with attention deficit hyperactivity disorder. *Int. J. Pediatr.* 13671–13681. 10.22038/IJP.2021.57015.4466

[B59] Sánchez-Meca J. Botella J. (2024). Moderators analysis in meta-analysis: Meta-regression and subgroups analyzes. *Cir. Esp.* 102 446–447. 10.1016/j.cireng.2024.03.012 39223006

[B60] Sibley M. H. Arnold L. E. Swanson J. M. Hechtman L. T. Kennedy T. M. Owens E. (2022). Variable patterns of remission from ADHD in the multimodal treatment study of ADHD. *Am. J. Psychiatry* 179 142–151. 10.1176/appi.ajp.2021.21010032 34384227 PMC8810708

[B61] Silva L. A. D. Doyenart R. Henrique Salvan P. Rodrigues W. Felipe Lopes J. Gomes K. (2020). Swimming training improves mental health parameters, cognition and motor coordination in children with attention deficit hyperactivity disorder. *Int. J. Environ. Health Res.* 30 584–592. 10.1080/09603123.2019.1612041 31081373

[B62] Sterne J. A. C. Savoviæ J. Page M. J. Elbers R. G. Blencowe N. S. Boutron I. (2019). RoB 2: A revised tool for assessing risk of bias in randomised trials. *BMJ* 366:l4898. 10.1136/bmj.l4898 31462531

[B63] Veronesi G. F. Gabellone A. Tomlinson A. Solmi M. Correll C. U. Cortese S. (2024). Treatments in the pipeline for attention-deficit/hyperactivity disorder (ADHD) in adults. *Neurosci. Biobehav. Rev.* 163:105774. 10.1016/j.neubiorev.2024.105774 38914177

[B64] Wang S. (2023). Yoga for emotional control in children with ADHD. *Rev. Bras. Med. Esporte* 29:e2022_0391. 10.1590/1517-8692202329012022_0391

[B65] Xie Y. Gao X. Song Y. Zhu X. Chen M. Yang L. (2021). Effectiveness of physical activity intervention on ADHD symptoms: A systematic review and meta-analysis. *Front. Psychiatry* 12:706625. 10.3389/10.3389/fpsyt.2021.706625 34764893 PMC8575983

[B66] Zhang M. Yu J. Li H. Jin R. Zhou J. Zhu T. (2025). Altered auditory attention and functional connectivity in the auditory cortex of children with attention-deficit/hyperactivity disorder. *BMC Psychiatry* 25:1033. 10.1186/s12888-025-07516-6 41163181 PMC12574008

